# Ecological niche modeling based on ensemble algorithms to predicting current and future potential distribution of African swine fever virus in China

**DOI:** 10.1038/s41598-022-20008-x

**Published:** 2022-09-16

**Authors:** Yue-peng Li, Xiang Gao, Qi An, Zhuo Sun, Hong-bin Wang

**Affiliations:** 1 College of Veterinary Medicine, Northeast Agricultural University, Harbin, People’s Republic of China; 2 Key Laboratory of the Provincial Education, Department of Heilongjiang for Common Animal Disease Prevention and Treatment, College of Veterinary Medicine, Northeast Agricultural University, Harbin, People’s Republic of China

**Keywords:** Ecological epidemiology, Computational biology and bioinformatics

## Abstract

African swine fever (ASF) is a tick-borne infectious disease initially described in Shenyang province China in 2018 but is now currently present nationwide. ASF has high infectivity and mortality rates, which often results in transportation and trade bans, and high expenses to prevent and control the, hence causing huge economic losses and a huge negative impact on the Chinese pig farming industry. Ecological niche modeling has long been adopted in the epidemiology of infectious diseases, in particular vector-borne diseases. This study aimed to establish an ecological niche model combined with data from ASF incidence rates in China from August 2018 to December 2021 in order to predict areas for African swine fever virus (ASFV) distribution in China. The model was developed in R software using the biomod2 package and ensemble modeling techniques. Environmental and topographic variables included were mean diurnal range (°C), isothermality, mean temperature of wettest quarter (°C), precipitation seasonality (cv), mean precipitation of warmest quarter(mm), mean precipitation of coldest quarter (mm), normalized difference vegetation index, wind speed (m/s), solar radiation (kJ /day), and elevation/altitude (m). Contribution rates of the variables normalized difference vegetation index, mean temperature of wettest quarter, mean precipitation of coldest quarter, and mean precipitation of warmest quarter were, respectively, 47.61%, 28.85%, 10.85%, and 7.27% (according to CA), which accounted for over 80% of contribution rates related to variables. According to model prediction, most of areas revealed as suitable for ASF distribution are located in the southeast coast or central region of China, wherein environmental conditions are suitable for soft ticks’ survival. In contrast, areas unsuitable for ASFV distribution in China are associated with arid climate and poor vegetation, which are less conducive to soft ticks’ survival, hence to ASFV transmission. In addition, prediction spatial suitability for future ASFV distribution suggests narrower areas for ASFV spread. Thus, the ensemble model designed herein could be used to conceive more efficient prevention and control measure against ASF according to different geographical locations in China.

## Introduction

African swine fever (ASF) is a hemorrhagic disease of domestic swine and wild boars with extremely high mortality rates (usually close to 100%)^1^, being considered a mandatory notifiable disease by the World Organization for Animal Health (OIE)^2,3^. Clinical manifestations of ASF are diverse, ranging from chronic and subclinical to hemorrhagic fever and sudden death, depending on the type of virus virulence and host susceptibility^3,4^.

African swine fever virus (ASFV) is a large, enveloped DNA virus, and the only member of the genus *Asfivirus* in the family *Asfaviridae*. ASFV is the only known tick-borne DNA arbovirus involving a complex cycle of transmission among swine, wild boars, and soft ticks^1,3^.

ASFV spreads in various ways. Soft ticks are the vectors and reservoirs of ASFV. Soft ticks have long survival time, and the virus can survive for up to five years when enters *Ornithodoros erraticus* ticks. In tick reservoirs, ASFV could be transmitted from male ticks to female ticks during mating, and female ticks in turn transmit the virus to the prole by infected oocytes. Soft ticks of the genus *Ornithodoros*, including *Ornithodoros moubata* complex ticks and *Ornithodoros erraticus* soft ticks, are involved in the transmission of ASFV to domestic swine^5^. Thus, infected domestic swine spread the virus rapidly to the group via blood, excrement, and carcasses. Environmental transmission is also an important route of ASFV dissemination. Contaminated transportation vehicles can also have a role in transmission of ASFV, pork products may be a key factor in determining outbreaks and the spread of ASF in China^6–8^.

ASF was first described in Kenya, and subsequently spread worldwide thus resulting in a global pandemic. In 2017, ASF was then described in Siberia, Russia, and subsequently a large number of ASF outbreaks occurred in the Russian Far East^8^. African swine fever first emerged in Shenyang, China in August 2018, and then spread to many Chinses provinces, causing a nationwide outbreak. ASF had a negative impact on animal health and economic development in world. As the world's largest pork producer and consumer, China has been impacted by the ASF epidemic. At the same time, ASF has an important impact on the global economy and trade^4^. However, ASF might also accelerate the transformation of China's pig farming industry and propel better standardization and the development of safer production systems^7,8^.

Ecological niche models are employed to explore the relationship between the distribution range of species and corresponding environmental variables. The basis of this model is that the species-environment relationship can explain and predict present and future species spatio-temporal distribution^9^. Ecological niche model has been widely used in biology, wildlife protection, global biological change, and widely used in the field of epidemiology^10–12^. In addition, ecological niche models have been successfully applied to disease control and prediction. Cheng et al.^13^ compared the ecological niche model and the epidemiological model to assess the risk of Usutu virus transmission in Europe. The results showed, the ecological niche model could better describe disease distribution, whereas the SEIR model could correctly predict the area of disease spread, but not temporal disease dynamics, thus demonstrating the difference between the applicability of the ecological niche model and the epidemiological model.

With the democratization of information and access to information, environmental and topographic variables can be freely obtained, which can be combined with animal disease surveillance data, to establish ecological niche models of disease incidence and spatio-temporal suitability for virus transmission. The widespread use of R software has diversified the development of ecological niche models and provided a platform for the collection of multiple models^14^. A free R package for developing ensemble models, i.e., Biomod, was developed by Thuiller et al. in 2003^15–18^.

Thus, the present work aimed to evaluate the spatio-temporal suitability areas for ASFV spread by establishing an integrated ecological niche model based on ASF incidence data in China from August 1, 2018, to December 31, 2021, combined with biological environmental and topographic variables. And provide useful ideas for ASF prevention and control measures for different geographical locations in China. Besides, we predicted spatio-temporal ASFV spread and the future suitability areas for the current to 2040 and 2040 to 2060.

## Material and methods

### Epidemiological data collection

**Figure 1:**
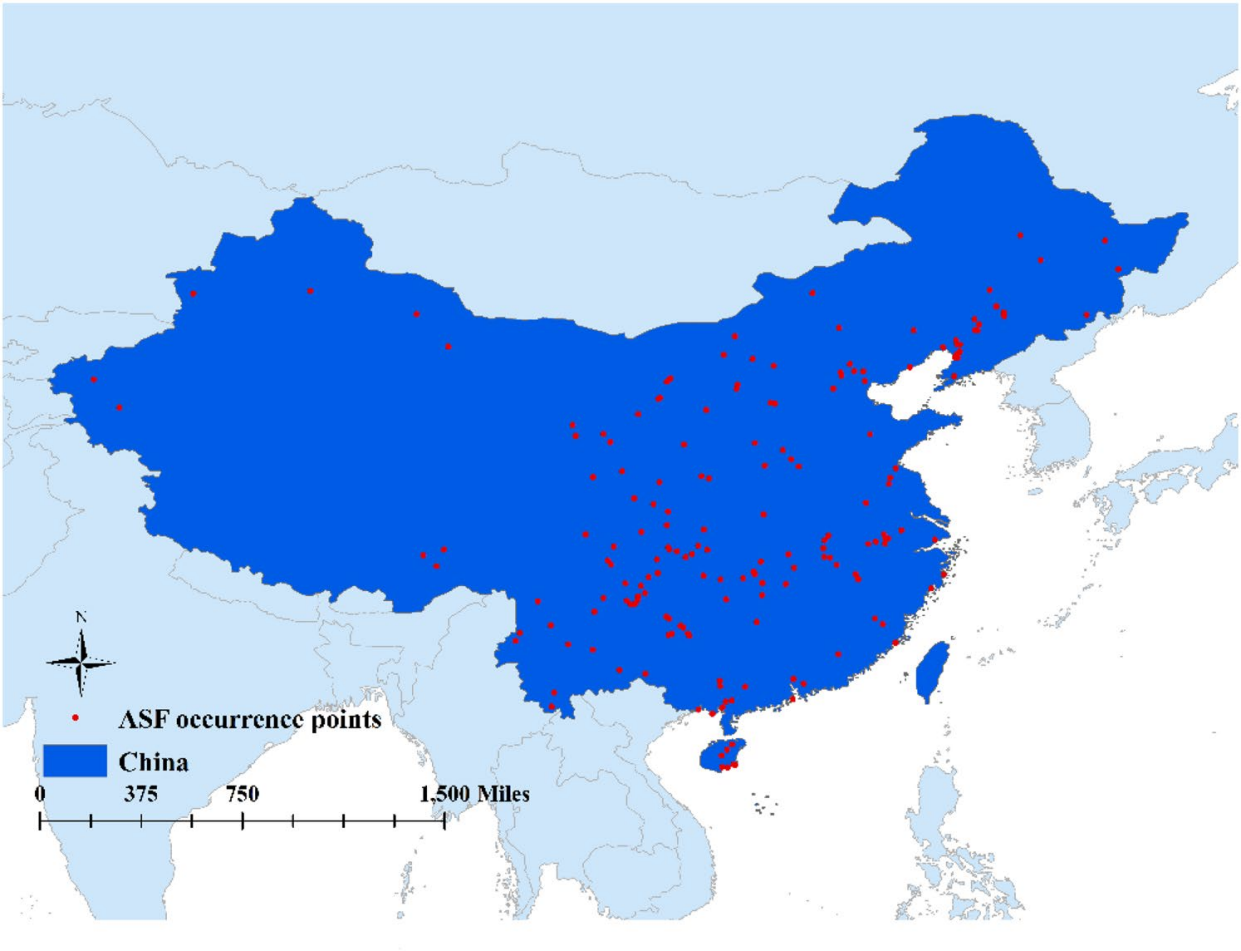
ASF occurrence point (The map is made by ArcGIS software and uses the WG-1984 coordinate system).

Data on ASF cases and outbreaks occurring in China from August 1, 2018, to December 31, 2021 were reported from 194 reports published by the World Organization for Animal Health (OIE) (https://www.oie.int/) and Food and Agriculture Organization of the United Nations (FAO) (https://empres-i.apps.fao.org/). Data collected included information about location, region, and specific latitude and longitude coordinates of reported ASF cases and outbreaks. To be included in the study, all points had to fall within the study area and cases records were checked for possible error. The R package ‘range Builder’(https://github.com/ptitle/rangeBuilder) was used for spatial point filtering to reduce spatial clustering, considering the filtering range 10 km in the R software. To build, the reduced spatial clustering model, 179 cases reports occurred in China were included (Fig. 1).

### Environmental and topographic variables collection

Environmental and topographic variables are important components of ecological niche models^19^. Topo-graphic and climate variables were downloaded from the Worldclim 2.1 database (https://worldclim.org).

Environmental and topographic variables were collected, which included 19 Bioclimatic variables, mean annual maximum temperature, mean annual minimum temperature, mean annual precipitation, solar radiation, elevation, wind speed, average temperature, water vapor pressure, and normalized difference vegetation index (NDVI). NDVI from China Resource and Environment Science and Data Center (https://www.resdc.cn/data.aspx?DATAID=257). 19 Bioclimatic variables, 6 environmental variables, and NDVI were for the ecological niche model designed herein (supplementary material Table 1). Besides, projected bioclimatic datasets from 2020 to 2040 and 2040 to 2060 were downloaded from the new WorldClim 2.1 database (https://worldclim.org).

**Table 1:** performance of a single model used KAPPA, TSS, and ROC.

Evaluation	GLM	GBM	GAM	CTA	ANN	SRE	FDA	MARS	RF	Maxent
KAPPA	0.63	0.73	0.82	0.62	0.49	0.73	0.62	0.71	0.79	0.87
AUC	0.92	0.97	0.98	0.92	0.86	0.84	0.91	0.92	0.95	0.95
TSS	0.78	0.82	0.90	0.8	0.62	0.68	0.71	0.75	0.94	0.83

### Environmental variables processing

All variables were in ESRI Grid raster file format. ArcGIS software was used to trim raster data to obtain same extent, cell size (2.5 arcmins of resolution), and coordinate system. Trimmed raster data were restricted to China extent using China map layers in shapefile format. However, due to multicollinearity, these variables could not all be included in the model. Multicollinearity checks were performed on 26 environmental variables which were verified using the VIF procedure of the USDM package in R software^20^. Variables with Pearson correlation coefficient greater than 0.7 and VIF values greater than 8 were excluded from the variable set. 10 variables were finally retained for use in the model (refer to supplementary material Table 2).

**Table 2:** evaluation for ensemble models use KAPPA, TSS, and ROC.

Evaluation	Mean suitability	CA	Weight mean
KAPPA	0.9	0.92	0.91
AUC	0.94	0.92	0.94
TSS	1.0	0.99	1.0

### Model establishment

Models were established using R software. Biomod 2 is a widely used R package for building ensemble models since it is freely available and offers diverse algorithms^21–23^. Thus, the Biomod 2 package was used to establish a predictive model to describe environmental suitability areas of ASFV distribution in China. The ensemble model is the pool of ten algorithms, namely Artificial Neural Networks (ANN), Surface Range Envelope (SRE), Flexible Discriminant Analysis (FDA), General Linear Models (GLM), General Additive Models (GAM), General Boosted Models (GBM), Classification Tree Analysis (CTA), Multiple Adaptive Regression Splines (MARS), Random Forests (RF), and Maximum Entropy model (Max Ent)^17^. Except Max Ent, and other models employ a presence-absence approach^24^, a surface range envelope model was used to generate 1000 absence points^25,26^. Pseudo-absence points selected by SRE improved model prediction sensitivity.

In the model ensemble process, GLM, GBM, GAM, and RF algorithms required adjusted arguments. Under the GLM function, the formula given was defined as quadratic and the information criteria for the stepwise selection procedure was AIC; Decision tree arguments were 500 in GBM function; GAM function was defined as "GAM_mgcv"; parameter of "mtry" was 3 in RF function.

A model option in Biomod 2, each algorithm was run three times, for a total of thirty runs for the ten algorithms. The data sets were divided in two parts: 80% of data was used to develop model, and 20% of data was used to evaluation model. The Area Under the Receiver Operating Curve (AUC) ROC curve, KAPPA, and the true skill statistic (TSS) were used to evaluate the performance of single and ensemble models^27^. Ensembles with a TSS below 0.8 were not included in the model. Variables included to build ensemble models were mean suitability, weighted mean, and committee averaging (CA) to represent global suitable level. CA has been incorporated in the ensemble modeling approach. First, it could be used to predict a suitable niche model, and second, it could also be used to evaluate the performance of the model^20^.

The CMIP5 climate model adopted in the IPCC5 Assessment Report, whereas the CMIP6 model adopted the latest assessment report. Compared with the previous climate model, the CMIP6 adds four Shared Socioeconomic Pathways (SSP), forming four new scenarios, i.e., SSP1-2.6, SSP2-4.5, SSP4-6.0, and SSP5-8.5. Compared to RCP4.5, SSP2-4.5 had a higher starting point and a slightly slower and relatively mild decline. SSP4-6.0 differed significantly from RCP6.0 in that CO_2_ emissions will peak and decline after 2050 compared to 2080. This mitigation strategy compensates higher short-term emissions, although greenhouse gas emissions other than carbon dioxide also played an important role. Instead, the CO_2_ emissions of SSP5-8.5 are significantly higher than those of RCP8.5, with correspondingly larger reductions in non-CO_2_ emissions. Considering that China was the focus of the current research the SSP2-4.5 scenario was adopted under the BCC-CSM2-MR model as the predicted climate variable^28–30^.

The suitability map was output after the completion of model prediction, and the map was defined as a Geo-Tiff raster format using the ArcGIS software. The suitability values of CA, mean suitability, and weight mean range of 10 to 1000 were obtained when classified by continuous linear values in the ArcGIS software. Each value was divided by 1000 to classify fitness values into the following five components, 0 (not suitable), 0.25 (moderately suitable), 0.5 (relatively suitable), 0.75 (suitable) and 1 (very suitable)^31^.

## Results

### Evaluation of ensemble models and impact of chosen variables

Table 1 depicts average values of KAPPA, TSS, and ROC of the thirty models designed in the present study. Considering individual models, the RF model had the best performance (TSS = 0.94, AUC = 0.95), followed by GAM (TSS = 0.98, AUC = 0.90) and the Maxent model (TSS = 0.83, AUC = 0.95). The worst performance was verified with ANN and SRE as denoted by TSS (0.62and 0.68, respectively). Based on the thirty models evaluated, we selected those with a TSS > 0.8 for integration to obtain the ensemble model. KAPPA, TSS, and ROC values of ensemble model were determined using mean suitability, CA, and weighted mean. The results indicated that the ensemble model phenotype had an outstanding performance. (Table 2).

Subsequently, future ten variables were included in ecologic niche models for prediction. In individual models, the variable with the highest contribution rate was NDVI (42.19%), followed by mean temperature of wettest quarter (35.21%), mean precipitation of warmest quarter (25.85%), and elevation/altitude (19.94%); the contribution rates of other variables are reported in the supplementary material (supplementary material Table 3). In the ensemble model, the contribution rate of NDVI was 47.61%, whereas the contribution rate of mean temperature of wettest quarter was 28.85%, mean precipitation of coldest quarter and mean precipitation of warmest quarter were 28.85%, 10.85%, and 7.27%, respectively (according to CA), and contribution rates of other variables are reported in Table 3. The normalized difference vegetation index, mean temperature of the wettest quarter, mean precipitation of coldest quarter, and mean precipitation of warmest quarter were the most important variables in the model prediction.

**Table 3:** 10 variables importance in the ensemble model.

Variable code	Mean suitability	CA	Weighted mean
bio_2	2.71	2.86	2.65
bio_3	3.02	2.76	3.27
bio_8	23.57	28.85	24.09
bio_15	2.40	1.72	2.61
bio_18	9.10	7.27	10.27
bio_19	8.20	10.85	9.28
NDVI	39.84	47.61	38.01
Wind	1.67	2.06	1.81
Srad	3.10	2.67	3.33
Elev	5.96	4.51	5.92

### Predicting suitability areas for ASF in China

**Figure 2:**
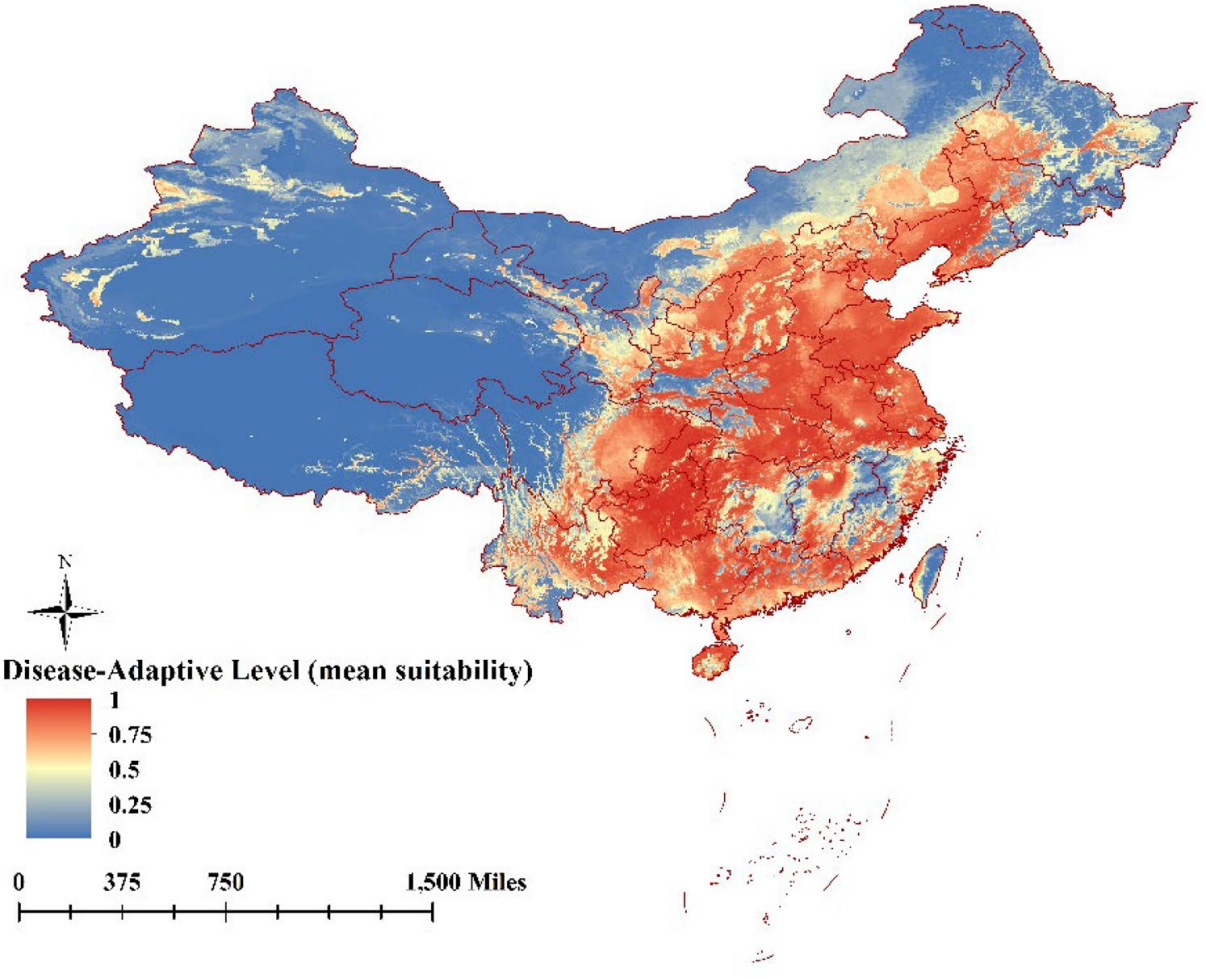
The disease-adaptive level of the ensemble model was expressed as the mean suitable. (The map is made by ArcGIS10.2 https://www.esri.com/ and R 4.1.3 software https://mirrors.bfsu.edu.cn/CRAN/).

**Figure 3:**
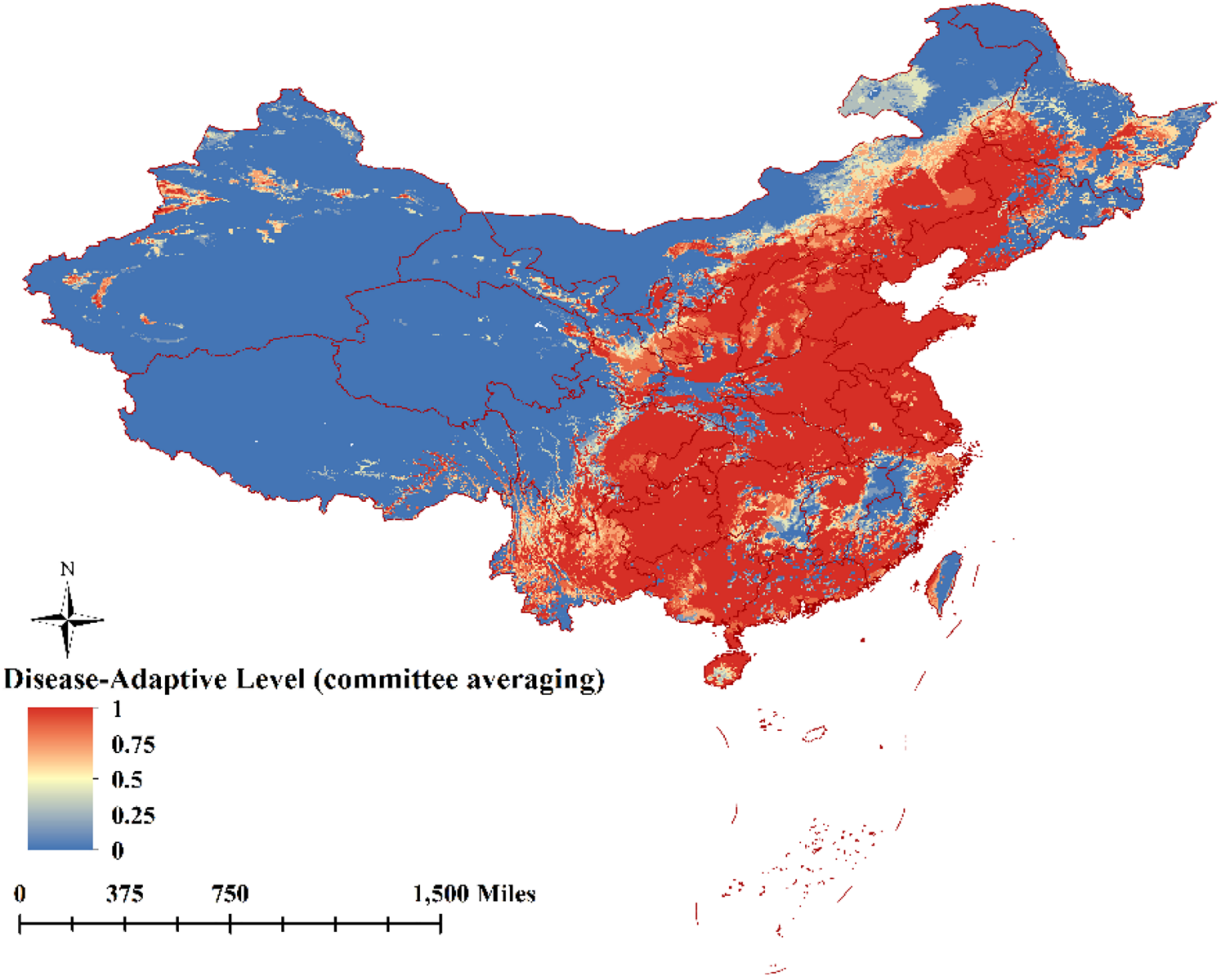
The disease-adaptive level of the ensemble model was expressed as the committee averaging (The map is made by ArcGIS10.2 https://www.esri.com/ and R 4.1.3 software https://mirrors.bfsu.edu.cn/CRAN/).

**Figure 4:**
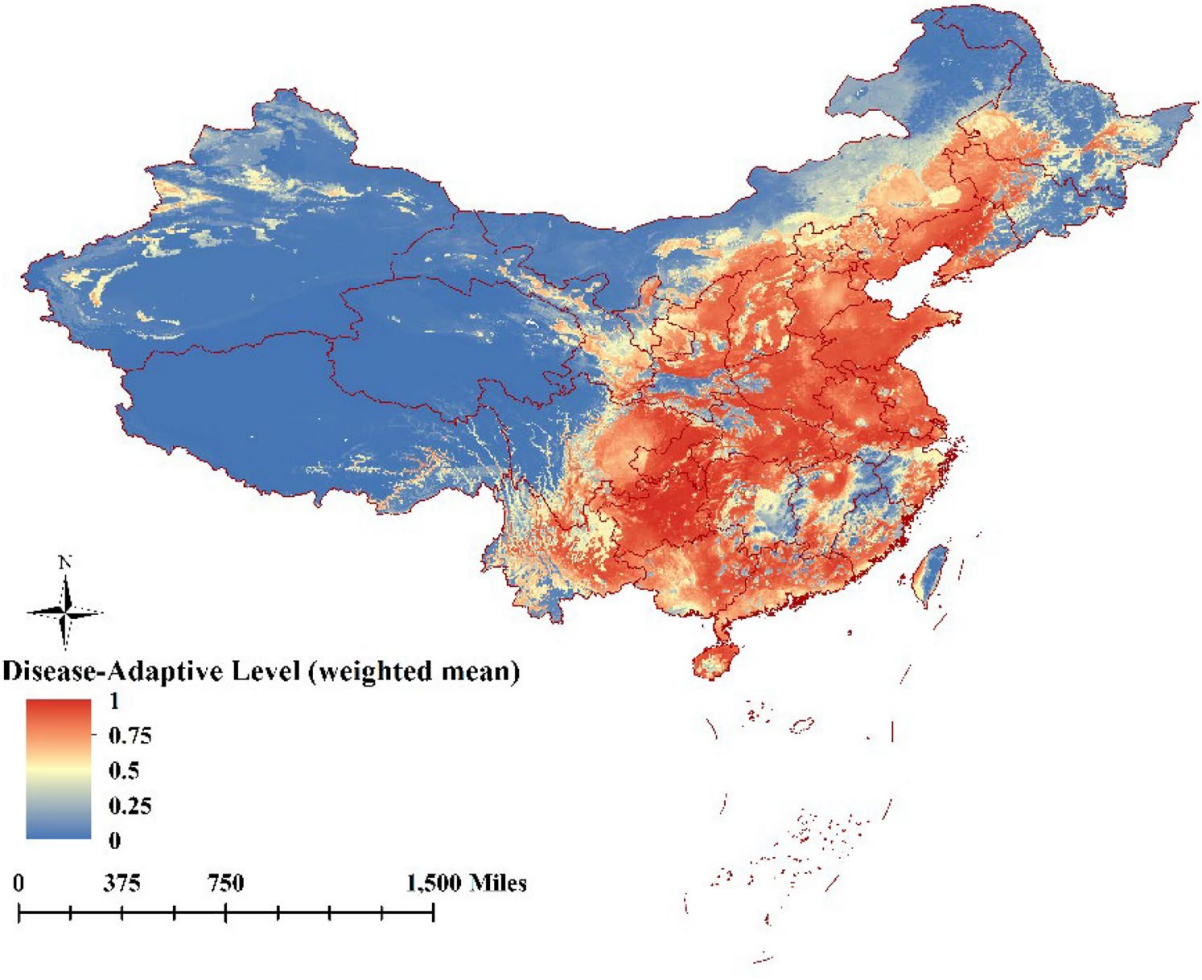
The disease-adaptive level of the ensemble model was expressed as the weighted mean suitable (The map is made by ArcGIS10.2 https://www.esri.com/ and R 4.1.3 software https://mirrors.bfsu.edu.cn/CRAN/).

Disease-adaptive level of the ensemble model was expressed as a function of the variables mean suitability level (Fig. 2), committee averaging (Fig. 3), and weighted mean suitability level (Fig. 4). That the very suitable areas for ASFV transmission was located on the southeast coast or central region of China, in particular the southern parts of Liaoning, Hebei, Shandong, Jiangsu, Anhui, Hunan, Hubei, Chongqing, and Guizhou provinces; the southern parts of the Inner Mongolia Autonomous Region, and Ningxia, Shaanxi, and Shanxi provinces, the eastern parts of Sichuan, Zhejiang provinces, the coastal areas of Fujian, and Guangdong, Guangxi provinces, and most area of Yunnan. Xinjiang, Qinghai, Tibet, and the northwestern parts of Inner Mongolia Autonomous Region and Gansu province were considered unsuitable for ASFV transmission. The predictions of the model for determining suitability of areas for ASFV transmission were broadly consistent with collected data concerning the regions where the disease has been previously reported (Fig. 1).

### Model uncertainty

**Figure 5:**
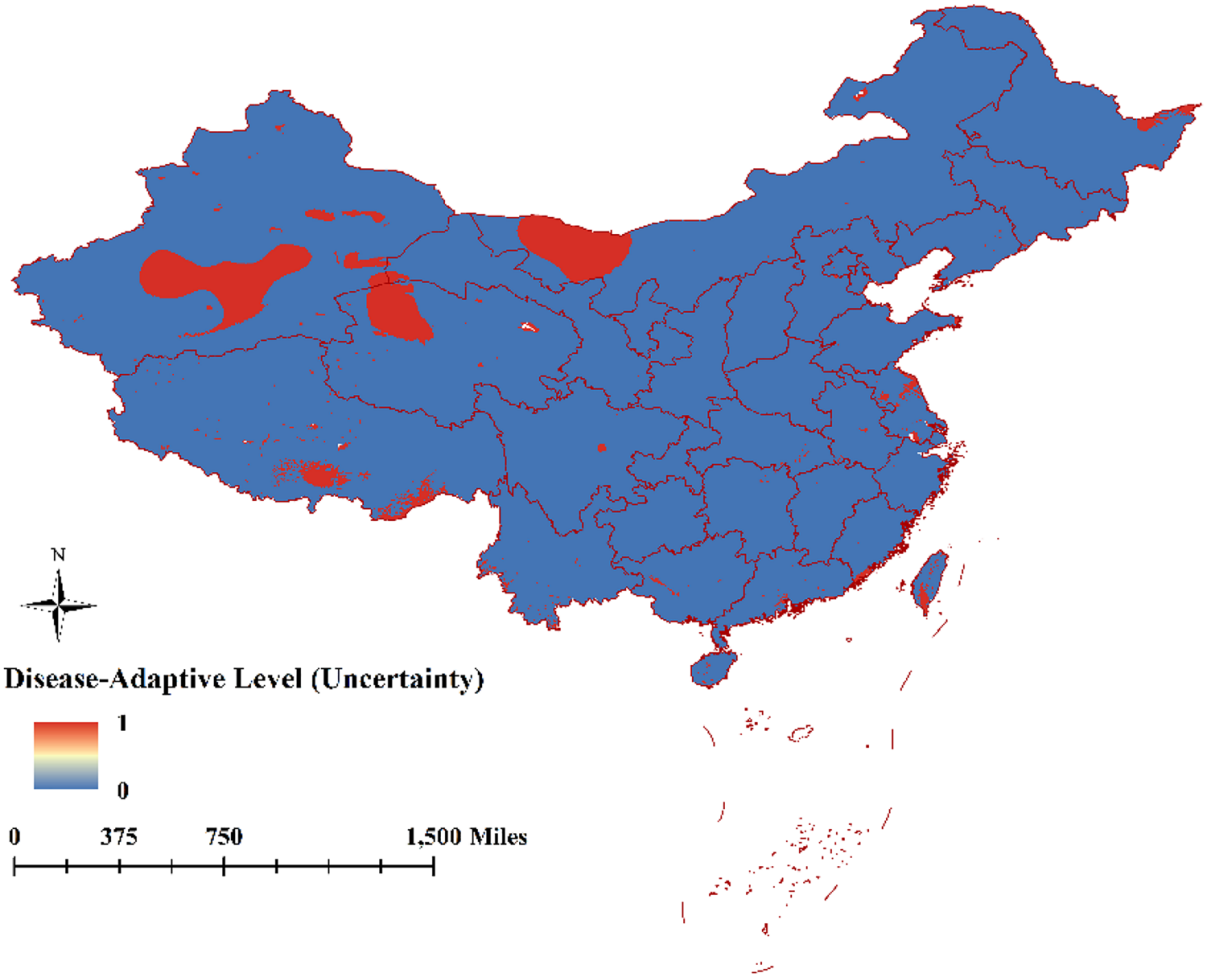
Model uncertainty was represented by clamping mask value. 1 (red) represents the uncertainty of the prediction model, 0.5 means that half of the model was certain and a half was uncertain, and 0 (blue) indicates that the model prediction was deterministic (The map is made by ArcGIS10.2 https://www.esri.com/ and R 4.1.3 software https://mirrors.bfsu.edu.cn/CRAN/).

The uncertainty of the model was measured by mask clamping (Fig. 5). There are only two corresponding values 1 (red) and 0 (blue) in the figure. When the value is closer to 1, it represents the uncertainty of the model prediction. When the value is 0.5, it means that half of the model prediction is certain, and half is uncertain. When the value is 0, the model prediction is accurate (27). The clamping mask results indicated high uncertainty in the central part of autonomous region of Xinjiang, the northwest of Qinghai province, and a small part of Inner Mongolia Autonomous Region. (Supplementary material Fig. 1).

### Prediction of future suitable areas for ASFV transmission

**Figure 6:**
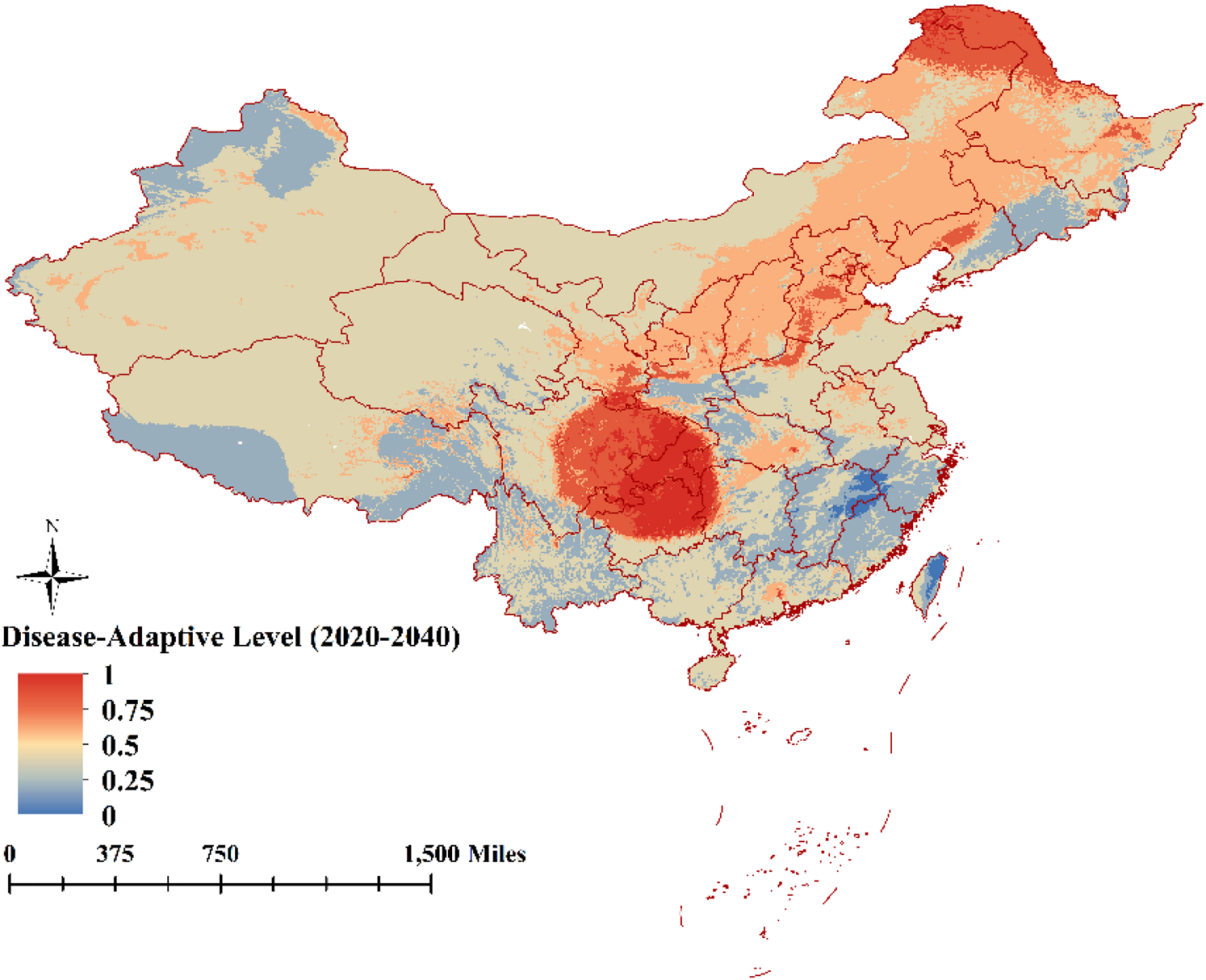
The model predicts areas suitable for ASF from 2020 to 2040 (The map is made by ArcGIS10.2 https://www.esri.com/ and R 4.1.3 software https://mirrors.bfsu.edu.cn/CRAN/).

**Figure 7:**
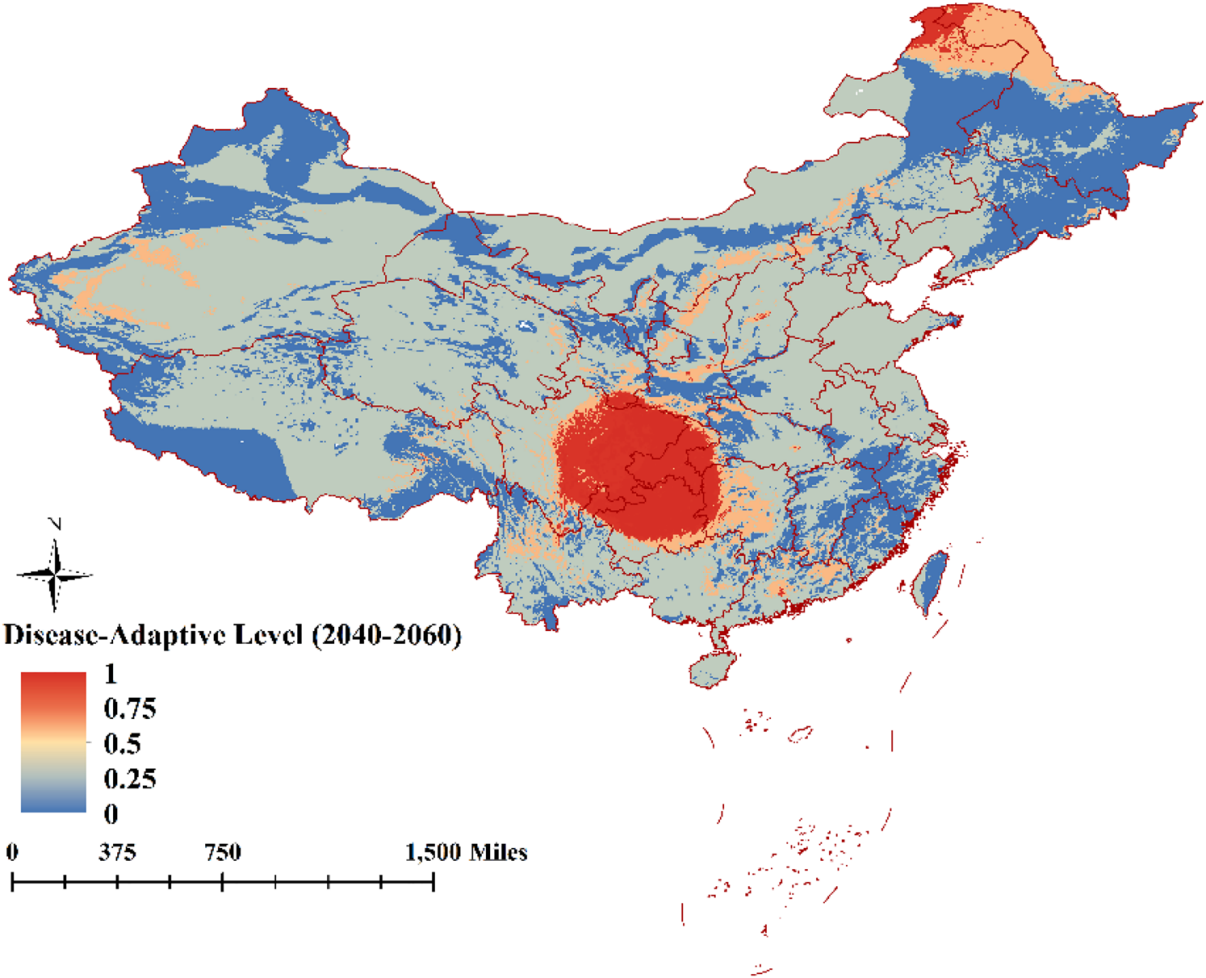
The model predicts areas suitable for ASF from 2040 to 2060 (The map is made by ArcGIS10.2 https://www.esri.com/ and R 4.1.3 software https://mirrors.bfsu.edu.cn/CRAN/).

The present study forecasted fluctuations in distribution suitability areas of ASFV transmission in China for the periods 2020 to 2040 and 2040 to 2060. Predictions for suitability area for ASFV transmission indicated a significant reduction in 2040 and 2060. However, general spatial suitability for ASFV transmission in 2040 will transfer to the central and northeast regions of China according to the model predictions, with the very suitability for ASFV transmission in the area of provinces Sichuan, Guizhou, and Chongqing (Fig. 6). By 2060, spatial suitability for ASFV transmission will be further reduced, and the very suitable will the few areas of Sichuan, Guizhou, and Chongqing (Fig. 7).

## Discussion

ASFV mainly infects wild boars and domestic swine, with soft ticks as its main vectors of transmission^5^. In particular, it is known that ASFV carried by wild boars can be transmitted to domestic swine by soft ticks^32^. Despite current efforts on ASF vaccine research, no commercial vaccine is currently available on the market yet^33,34^. Thus, measures for prevention and control for ASF outbreaks are still necessary. ASF transmission occurs in three independent epidemiological cycles: sylvatic, tick-pig, and domestic swine, those cycles including soft ticks, wild boars (especially warthog), domestic swine, and porcine-derived products^35^. The sylvatic cycle mainly occurs in Africa^5^; in the tick-pig cycle, soft ticks act as hosts, allowing ASFV to persist in the environment^32,36^.

African swine fever is the most significant disease threat to swine globally, and ASF were constantly being reported in various countries. In March 2017, ASF emerged in Irkutsk, southeastern Siberia, Russian^37^ and in August 2018, ASF was described in Shenyang, China^4^, which showed that ASFV can be spread across long geographical distances, probable as a result of human activity^35^.

It is well known that the ecological niche models have been widely used in the field of epidemiology, especially for arbovirus as well as endemic, and direct contact diseases^10^. The ensemble model avoids the shortcomings of a single algorithm to a certain extent, and increases the accuracy of the model's prediction. In addition, the predictive power of the ensemble model was significant than that of every individual models. In the present study, we developed an ensemble model (TSS = 1, AUC = 0.94, KAPPA = 0.9), and proved that the model could accurately predict the spatial suitability and unsuitable areas for ASFV transmission.

Contribution rates of variables of normalized difference of vegetation index, mean temperature of wettest quarter, mean precipitation of coldest quarter, and mean precipitation of warmest quarter were, respectively, 47.61%, 28.85%, 10.85%, and 7.27% (according to CA); and these four variables accounted for over 80% of contribution rates related to variables. Temperature and humidity can affect the survival and transmission rate of viruses in the environment. Persistence of dried infectious ASFV on paper are significantly the longest at lower environmental temperatures whereas that of dried infectious ASFV on paper is significantly the shortest at higher environmental temperature, but that limited by the type of pollutants and ambient humidity^38^. ASFV in feces could remain infectious longer in 4 °C than 37 °C^39^. Soil type also affects ASFV stability, and stability of infectious ASFV is very low in acidic forest soils but rather high in sandy soils^40^. Distribution of vegetation, rainfall frequency and abundance, and temperature are all important factors affecting arthropods distribution. In addition, survival of soft ticks is strongly related to seasonality. Moreover, temperature and humidity affect hatching rate of soft ticks. Generally, distribution occurs at higher rates in spring and autumn, and lower rates in summer and winter^41,42^. Although the variable soft tick distribution was not included in present study, distribution suitability for soft ticks in China described by Liu Jian et al.^43^ largely overlaps with suitability for ASFV distribution in China described herein.

In addition, most areas revealed herein as suitable for ASFV distribution are located along the southeast coast or central region of China, wherein warm climate, abundant rainfall, and rich vegetation are conditions suitable for soft ticks’ survival and multiplication^35^. The large-scale pig breeding in these areas, the irregular operation of relevant practitioners, example the lack of thorough disinfection and personnel isolation after contact with ASFV-infected pigs were also one of the important reasons for the spread of the disease. Large-scale and intensive pig farming in Guizhou, Ningxia, and other China provinces is still incipient, and most farming are family pig farming households. In such conditions, swill inevitably gets mixed and may be contaminated by viruses, and intensive contact in pig herds increases risk of direct transmission, which might explain why such regions could easily be implicated in ASF outbreaks^37^. In addition, the provinces Xinjiang, Qinghai, and Tibet, as well as the northwestern parts of the Inner Mongolia Autonomous Region and Gansu were shown to be unsuitable for ASFV transmission. These areas have relatively arid climate, and poor vegetation, which are less conducive to soft ticks’ survival, was unsuitable for ASFV transmission. At the same time, these provinces do not have large-scale pig breeding, which also reduces the risk of ASF transmission. The prediction of the model designed in the present study was broadly consistent with data from areas where ASF is commonly being reported, which indicates that the model could predict the suitable distribution areas of ASF well.

The predicted results for periods 2020 to 2040 and 2040 to 2060 showed that the suitability areas for ASF in China will be reduced and transfer to the central and northeast regions of China, especially in 2060, and areas with highest susceptibility will be only the coastal area of Sichuan, Guizhou, and Chongqing. With the future climate change, the suitable distribution area of ASF was also decreasing, which is beneficial to the control of African swine fever. However, the most prone areas should continue to take reasonable prevention and control measures.

Nevertheless, certain uncertainties were still identified in the ensemble model designed in the present study. The ensemble model generally provides more accurate prediction compared to individual models, and the need to determine which algorithm offers a more accurate prediction is superfluous. However, studies have shown that the ensemble model may not result in better performance when compared to the individual models. Nevertheless, the ensemble model combines the advantages of a single algorithm with reduced noise^44,45^. In the present study, the ensemble model outperformed the individual model and successfully described the distribution suitability for ASF. However certain limitations were also observed in the designed ensemble model, e.g., a missed outbreak report. Since ASF can spread geographically, future studies should assess whether cases or outbreaks of ASF occurring in countries surrounding China might influence ASF distribution in China in the future. Furthermore, the potential impact of pseudo-missing values established using SRE on prediction results must be assessed. Thus, these limitations of the model must be taken into consideration.

## Conclusions

The model designed herein used bioclimatic factors to predict spatial suitability areas for ASF distribution in China, and could accurately distinguish between areas which will be suitable and unsuitable for ASF distribution. Normalized difference vegetation index, mean temperature of wettest quarter, mean precipitation of coldest quarter, and mean precipitation of warmest quarter variable contribution rate were most high. Most areas revealed herein as suitable for ASF distribution are located in the southeast coast or central region of China, wherein environmental conditions are suitable for soft ticks’ survival. In contrast, areas unsuitable for ASFV distribution in China as demonstrated by the ensemble model designed herein are associated with arid climate and poor vegetation which are less conducive to soft ticks’ survival, hence to ASFV transmission. Collectively, the ensemble model designed herein could identify with great accuracy locations more suitable for ASFV transmission in China, being thus useful for conceiving more effective preventive and control measures against ASF spread in China.

## Supplementary Information

Supplementary Information.

## Data Availability

The datasets generated during and/or analysed during the current study are available from the corresponding author on reasonable request.
