# Supplementary material for: Ecological niche modeling based on ensemble algorithms to predicting current and future potential distribution of African swine fever virus in China
Source: Sci Rep. 2022 Sep 16;12:15614. doi: 10.1038/s41598-022-20008-x (PMC9481527; doi:10.1038/s41598-022-20008-x)
Supplement: Supplementary file 1 — Supplementary Information. [file 41598_2022_20008_MOESM1_ESM.pdf]

Ecological niche modeling based on ensemble algorithms to predicting current and future potential distribution of African swine fever virus in China

Yue-peng Li[1,2]†, Xiang Gao[1,2], Qi an[1,2], Zhuo sun[1,2], Hong-bin Wang*[1,2]

1 College of Veterinary Medicine, Northeast Agricultural University, Harbin, People's Republic of China

2 Key Laboratory of the Provincial Education Department of Heilongjiang for Common Animal Disease Prevention and Treatment, College of Veterinary Medicine, Northeast Agricultural University, Harbin, People's Republic of China

Table 1. Shows 26 variables' Variable codes, names, and sources

Variable Code	Variable Name	Source
bio_1	Annual Mean Temperature (°C)	WorldClim version 2.1
bio_2	Mean Diurnal Range (°C)	WorldClim version 2.1
bio_3	Isothermality (BIO2/BIO7) (×100)	WorldClim version 2.1
bio_4	Temperature Seasonality (standard deviation ×100)	WorldClim version 2.1
bio_5	Max Temperature of Warmest Month (°C)	WorldClim version 2.1
bio_6	Min Temperature of Coldest Month (°C)	WorldClim version 2.1
bio_7	Temperature Annual Range (BIO5-BIO6) (°C)	WorldClim version 2.1
bio_8	Mean Temperature of Wettest Quarter (°C)	WorldClim version 2.1
bio_9	Mean Temperature of Driest Quarter (°C)	WorldClim version 2.1
bio_10	Mean Temperature of Warmest Quarter (°C)	WorldClim version 2.1
bio_11	Mean Temperature of Coldest Quarter (°C)	WorldClim version 2.1
bio_12	Annual Precipitation (mm)	WorldClim version 2.1
bio_13	Precipitation of Wettest Month (mm)	WorldClim version 2.1
bio_14	Mean Precipitation of Driest Month(mm)	WorldClim version 2.1
bio_15	Precipitation Seasonality (CV)	WorldClim version 2.1
bio_16	Precipitation of Wettest Quarter (mm)	WorldClim version 2.1
bio_17	Precipitation of Driest Quarter (mm)	WorldClim version 2.1
bio_18	Mean Precipitation of Warmest Quarter(mm)	WorldClim version 2.1
bio_19	Mean Precipitation of Coldest Quarter (mm)	WorldClim version 2.1
Tmax	Mean Annual Maximum Temperature (°C)	WorldClim version 2.1
Tmin	Mean Annual Minimum Temperature (°C)	WorldClim version 2.1
Prec	Mean Annual Precipitation (mm)	WorldClim version 2.1
Srad	Solar radiation (kJ /day)	WorldClim version 2.1
Elev	Elevation/Altitude (m)	WorldClim version 2.1

Wind	Wind Speed (m/s)	WorldClim version 2.1
Tavg	Average temperature (°C)	WorldClim version 2.1
Vapr	water vapor pressure (kPa)	WorldClim version 2.1
NDVI	Normalized Difference Vegetation Index	Resource and Environment Science and Data Center

Table 2. 10 variables used in the ensemble model.

Variable code	Variable name	Source
bio_2	Mean Diurnal Range (°C)	WorldClim version 2.1
bio_3	Isothermality (BIO2/BIO7) (×100)	WorldClim version 2.1
bio_8	Mean Temperature of Wettest Quarter (°C)	WorldClim version 2.1
bio_15	Precipitation Seasonality (CV)	WorldClim version 2.1
bio_18	Mean Precipitation of Warmest Quarter(mm)	WorldClim version 2.1
bio_19	Mean Precipitation of Coldest Quarter (mm)	WorldClim version 2.1
NDVI	Normalized Difference Vegetation Index	Resource and Environment Science and Data Center
Wind	Wind Speed (m/s)	WorldClim version 2.1
Srade	Solar radiation (kJ /day)	WorldClim version 2.1
Elev	Elevation/Altitude (m)	WorldClim version 2.1

Table 3. Variable importance for a single model

Variable Code	GLM	GBM	Maxent	GAM	CTA	ANN	SRE	FDA	MARS	RF	Mean
bio_2	4.80	2.65	17.40	11.11	0.00	24.15	34.43	6.63	7.18	4.30	11.26
bio_3	6.45	0.67	8.63	16.38	5.13	19.23	13.23	4.30	6.15	1.33	8.15
bio_8	62.85	34.73	42.68	22.63	53.30	26.58	27.48	12.33	57.60	11.93	35.21
bio_15	7.35	0.03	13.23	35.40	0.00	5.63	10.83	0.00	4.25	0.95	7.77
bio_18	44.78	3.93	28.25	56.78	1.05	53.53	21.80	23.55	16.75	8.08	25.85
bio_19	9.37	7.18	14.83	29.63	7.50	9.08	30.75	16.23	10.20	4.73	13.95
NDVI	50.45	45.73	46.18	61.23	71.33	0.10	55.43	28.90	42.00	20.60	42.19
Wind	0.00	0.45	12.15	6.98	5.73	2.30	14.10	1.33	0.00	3.40	4.64
Srad	8.58	0.25	17.08	12.48	0.00	9.30	4.35	47.25	1.23	3.35	10.39
Elev	7.75	0.55	8.25	57.33	0.55	88.30	24.78	0.00	5.40	6.45	19.94

Mapping Uncertainty

Effective use of ecological niche models relies on accurate presentation of uncertainty. The model does not perform equally well across space, and some hotspots of uncertainty may be important for future investigations and regional mapping assessments. The clamping mask value is

used in the article to represent the uncertainty of the model, and the coefficient of variation is supplemented here to further illustrate the uncertainty of the model. It can be observed in the figure that the areas with the highest model uncertainty are located in northwestern China and these areas are not suitable for ASFV distribution.

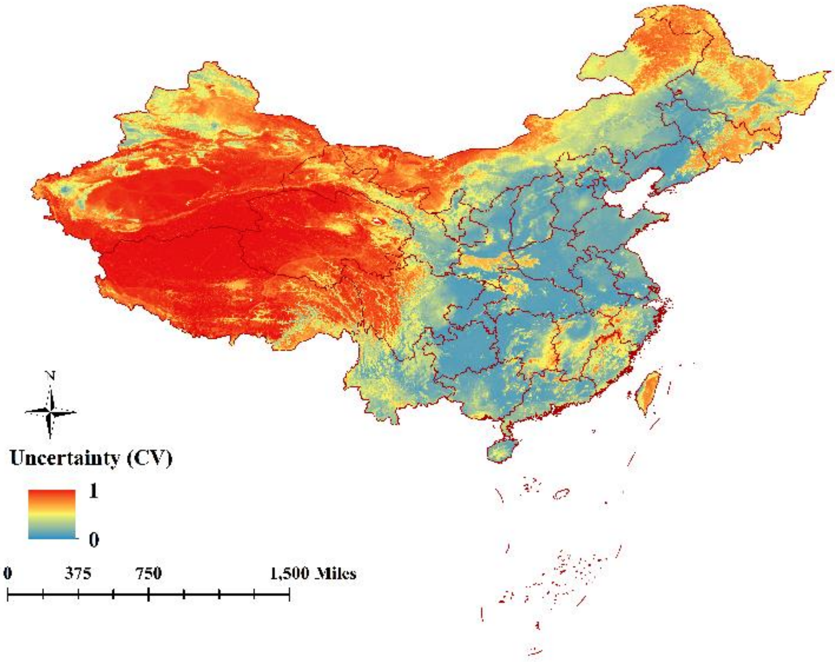

Supplementary Figure1: Uncertainty in the prediction of ASFV of represented by coefficient of variation. (The map is made by ArcGIS10.2 https://www.esri.com/ and R 4.1.3 software https://mirrors.bfsu.edu.cn/CRAN/)
